# HLA Genotype Imputation Results in Largely Accurate Epitope Mismatch Risk Categorization Across Racial Groups

**DOI:** 10.1097/TXD.0000000000001639

**Published:** 2024-06-20

**Authors:** Gregory S. Cohen, Alison J. Gareau, Melissa A. Kallarakal, Tayyiaba Farooq, Maria P. Bettinotti, H. Cliff Sullivan, Abeer Madbouly, Scott M. Krummey

**Affiliations:** 1 Department of Pathology, Johns Hopkins University School of Medicine, Baltimore, MD.; 2 Johns Hopkins Immunogenetics Laboratory, Baltimore, MD.; 3 Department of Pathology and Laboratory Medicine, Emory University School of Medicine, Atlanta, GA.; 4 National Marrow Donor Program/Be The Match, Minneapolis, MN.; 5 Center for International Blood and Marrow Transplant Research, Minneapolis, MN.

## Abstract

**Background.:**

Biomarkers that predict posttransplant alloimmunity could lead to improved long-term graft survival. Evaluation of the number of mismatched epitopes between donor and recipient HLA proteins, termed molecular mismatch analysis, has emerged as an approach to classify transplant recipients as having high, intermediate, or low risk of graft rejection. When high-resolution genotypes are unavailable, molecular mismatch analysis requires algorithmic assignment, or imputation, of a high-resolution genotyping. Although imputation introduces inaccuracies in molecular mismatch analyses, it is unclear whether these inaccuracies would impact the clinical risk assessment for graft rejection.

**Methods.:**

Using renal transplant patients and donors from our center, we constructed cohorts of surrogate donor-recipient pairs with high-resolution and low-resolution HLA genotyping that were racially concordant or discordant. We systemically assessed the impact of imputation on molecular mismatch analysis for cohorts of 180–200 donor-recipient pairs for each of 4 major racial groups. We also evaluated the effect of imputation for a racially diverse validation cohort of 35 real-world renal transplant pairs.

**Results.:**

In the surrogate donor-recipient cohorts, imputation preserved the molecular mismatch risk category for 90.5%–99.6% of racially concordant donor-recipient pairs and 92.5%–100% of racially discordant pairs. In the validation cohort, which comprised 72% racially discordant pairs, we found that imputation preserved the molecular mismatch risk category for 97.1% of pairs.

**Conclusions.:**

Overall, these data demonstrate that imputation preserves the molecular mismatch risk assessment in the vast majority of cases and provides evidence supporting imputation in the performance of molecular mismatch analysis for clinical assessment.

Organ transplantation is a life-saving therapy for patients with end-stage organ failure due to a wide range of diseases. Because of the tremendous population-level diversity of the HLA system, donors often express distinct HLA proteins from recipients, creating the potential for alloimmune responses that can result in tissue injury and graft failure.^1,2^ As a result, clinical testing is performed to ensure immunologic compatibility and avoid donor-specific HLA antibodies (DSAs) at the time of transplantation.^3,4^

Despite these measures, rejection and graft loss occur slowly but steadily in the years after transplantation.^5,6^ As an example, the formation of posttransplant or “de novo” donor-specific HLA antibody (dnDSA) occurs in approximately 20% of kidney transplant patients within the decade after transplantation, and it is a risk factor for graft loss.^1,6,7^ The formation of dnDSA after transplant is a risk factor for graft dysfunction or loss for heart and lung transplant patients.^8-10^ These findings have focused on identifying biomarkers that can predict the fraction of patients more likely to experience graft rejection despite the absence of DSAs at the time of transplant.^11,12^

In recent years, there has been increasing focus on the importance of HLA epitopes or biochemical sites recognized by HLA antibodies and shared across families of HLA proteins.^13,14^ Using HLA genotyping, the identity and number of mismatched epitopes can be evaluated, a process recently termed molecular mismatch analysis.^12^ Seminal work by Wiebe and Nickerson^13^ demonstrated that the magnitude of HLA class II (HLA-DR and HLA-DQB) epitope mismatches corresponded with the risk of the development of posttransplant DSAs and graft failure, a finding that has been verified in additional cohorts.^15-19^ These analyses have identified tiers of HLA molecular mismatches that correspond with high, medium, or low risk of posttransplant DSA formation.^15,20^ Although additional prospective and multicenter studies are ongoing, there is considerable interest in applying these concepts to organ allocation and clinical management of donor-recipient pairs.^12,13,21^

One potential barrier to using molecular mismatch analysis for clinical assessment is the need for high-resolution (eg, 2-field HLA-A*XX:YY) genotyping.^21-23^ Low-resolution genotyping (eg, 1-field, HLA-A*XX) is the standard for deceased donor transplantation due primarily to the required speed of testing results. To perform molecular mismatch analysis in these cases, the most likely allele-level genotype is identified or imputed on the basis of population studies and haplotype assessment.^22,24,25^ Thus, as the interest in performing molecular mismatch analysis in different patient cohorts grows, there is concern about the accuracy of performing this analysis on imputed HLA genotypes.^21,23,26,27^

Recent studies showed that imputation introduced multiple inaccuracies into HLA haplotype identification and suggested that this would impact molecular mismatch analysis. In particular, non-Caucasian racial groups were more impacted by imputation, which likely reflects the subtle differences in the depth of understanding of HLA genotypes at a population level.^23,28^ However, these studies did not comprehensively address the impact of imputation on the molecular mismatch risk classification, which is currently the best available tool for using this information in a clinical setting.^27^ In this study, we sought to gain insight into the impact of imputation on renal transplant patients across racial groups with high-resolution genotyping as a comparison using 2 sets of analysis. In the first cohort, we constructed surrogate donor-recipient pairs from each of 4 major racial groups to systematically compare racially concordant and discordant pairs. We then performed a similar analysis in a validation cohort of racially diverse renal transplant patients from our center.

## MATERIALS AND METHODS

### Study Design

To construct surrogate pairs, we identified renal transplant patients and donors who underwent high-resolution HLA genotyping for clinical management at Johns Hopkins Immunogenetics Laboratory from January 14, 2021, to June 30, 2022. The Johns Hopkins Institutional Review Board approved the study. Written informed consent was not obtained by study participants. This requirement was waived by the institutional review board. The clinical and research activities being reported are consistent with the Principles of the Declaration of Istanbul as outlined in the “Declaration of Istanbul on Organ Trafficking and Transplant Tourism.” High-resolution genotyping was performed by next-generation sequencing using the CareDx Alloseq Tx17 and Illumina sequencing platform to generate 2-field genotypes at 11 HLA loci. Patient and donor racial identities were collected from self-identified categories in the electronic medical record. Broad racial groups of Asian American, African American, Caucasian, or Hispanic are consistent with broad racial groups identified in US-based HLA allele and haplotype frequency studies.^25^ Individuals were labeled as donors or recipients using randomly generated identifiers for our analysis. For each racial group, 10 recipients and 20 donors were identified, except for Asian Americans, for which only 18 donors were available. To construct a renal transplant recipient cohort, we identified 35 consecutive kidney transplant patients transplanted at Johns Hopkins Hospital from March to June 2022 and performed high-resolution HLA genotyping by next-generation sequencing. To avoid caveats associated with transplantation between HLA-related individuals and highly HLA-matched unrelated pairs, we selected transplants performed from a deceased donor and patients who had a calculated panel-reactive antibody (cPRA) of ≤80.

### Imputation of High-resolution Data

HLA genotyping data were transformed to the serological/antigen-level low-resolution genotyping by removing the second HLA typing field, with the following exceptions based on serological splits, in which the second field is needed to assign a serological type: HLA-B*14, HLA-B*15, HLA-B*40, HLA-B*55, HLA-B*56, HLA-C*03, HLA-DRB1*03, HLA-DRB1*103, and HLA-DQB1*03. The low-resolution genotyping was imputed using the HaploStats algorithm (haplostats.org) to generate imputed high-resolution genotyping. For this analysis, only the most likely imputation result was considered for each input HLA genotype. Imputation was performed guided by the input self-identified race for each HLA sample.

Because HaploStats does not currently return a result for HLA-DQA1, HLA-DPB1, and HLA-DPA1 loci, the high-resolution genotyping for these loci was used for a subsequent analysis.

### Molecular Mismatch Analysis

For high-resolution and imputed surrogate cohorts, donor-recipient pairs were generated by matching each donor with all recipients within each racial group. For example, for Caucasians, 10 recipients were each paired with 20 donors to create 200 surrogate pairs. Using the hlaR package in R,^29^ the HLA Matchmaker algorithm was performed to quantify the molecular mismatches at each locus and assign the immunologic risk categorization.^15,20^ Molecular mismatch analysis was performed on the typed and imputed high-resolution HLA genotyping of identical pairs in parallel. The pairwise difference in the molecular mismatches between genotyping methods per HLA loci was calculated by the following formula, in which *m* is molecular mismatches, *x* is HLA loci, *H* is high resolution, and *I* is imputed:


$$\begin{array}{l} \;\Delta \;m=\left|m_{xH}-m_{xI}\right| \end{array}$$

The pairwise difference in molecular mismatches between genotyping methods for HLA class I and class II, respectively, were calculated using the following formulas:


$$\begin{array}{l} \;\Delta \;m_{classI}=\left|\left(m_{AH}+m_{BH}+m_{CH}\right)-\left(m_{AI}+m_{BI}+m_{CI}\right)\right| \end{array}$$

$$\begin{array}{ll} \;\Delta \;m_{classII} & =\left|(m_{DRB1H}+m_{DRB345H}+m_{DQB1H}\right)\;-\;\left(m_{DRB1I}+m_{DRB345I}+m_{DQB1I}\right)| \end{array}$$

### Statistical Analysis

The high-resolution and imputed cohorts generated were compared by the total number of epitope (molecular) mismatches, number of molecular mismatches by locus, changes in epitope mismatches, and changes in risk categorization. The number of epitope mismatches in the high-resolution and imputed cohorts were compared using the paired *t* tests. Simple linear regression was used to compare the number of high-resolution molecular mismatches and imputed molecular mismatches. Logistic regression was used to determine the odds of changes in the molecular mismatch risk categorization using imputation. All statistical analyses were performed using R (version 4.3.0).

## RESULTS

### Imputation Leads to Modest Changes in the Number of Molecular Mismatch at Each Individual HLA Locus

**FIGURE 1.**
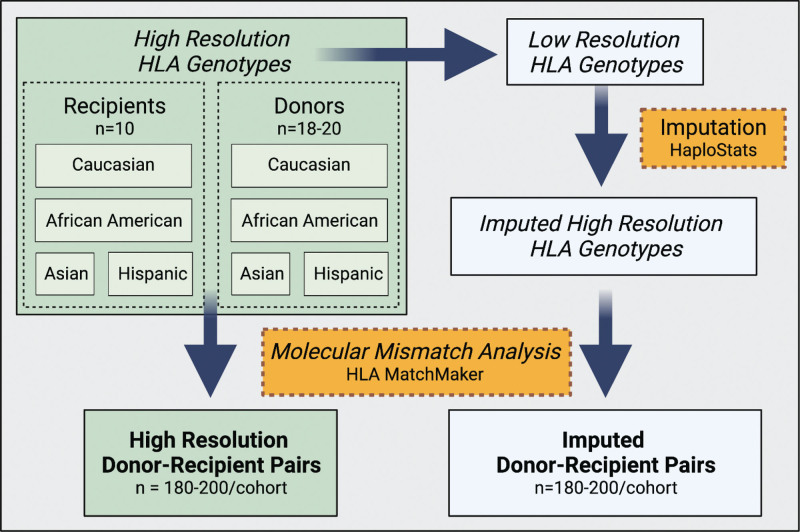
Schematic of the generation of surrogate donor-recipient kidney transplant pairs. Kidney transplant donors and recipients from each racial group with high-resolution HLA genotyping were selected. High-resolution genotyping was transformed into low-resolution and imputed using Haplostats. Molecular mismatch analysis of identical donor-recipient pairs was performed on the high-resolution or imputed HLA genotyping using HLA Matchmaker.

To investigate the impact of imputation on molecular mismatch analysis, we generated surrogate donor-recipient pairs based on renal transplant candidates or donors with high-resolution HLA genotyping at our institution. We identified groups based on self-identified racial groups (Figure 1). We took this approach to systematically identify the impact of imputation in racially concordant and discordant groups. We identified 10 recipients and 18–20 donors from each African American, Asian American, Caucasian, and Hispanic individuals to generate cohorts of 180–200 surrogate donor-recipient pairs. This number approximates the annual renal transplant volume of many US transplant centers. We transformed the HLA genotyping from high resolution to the serological antigen split level, or low resolution, which is the minimum requirement for HLA genotyping for deceased donor allocation. We then used HaploStats to impute the allele-level genotype. This provided us with parallel cohorts of definitive high-resolution HLA genotypes and imputed high-resolution genotypes to assess surrogate pairs using HLA Matchmaker (Figure 1). The selected imputation output from Haplostats was the most likely imputed genotypes within the same race group as the input sample. This was the imputed result used for all the analyses listed below.

We first evaluated the impact of imputation in changing the number of molecular mismatches at each HLA locus (class I: HLA-A, HLA-B, and HLA-C; class II HLA-DRB1, HLA-DRB345, HLA-DQB1) in surrogate pairs of the same donor-recipient race (Table 1). Overall, the number of class I molecular mismatches obtained by each method was highly correlated (*R*^2^ = 0.92–0.99 for African Americans, Caucasians, and Hispanics) and was lowest for Asian Americans (*R*^2^ = 0.88; **Figure S1A, SDC,** http://links.lww.com/TXD/A666). For each racially concordant cohort, the average number of single HLA locus molecular mismatches that were changed by imputation was small, with averages ranging from 0 to 2.39 molecular mismatches. For Caucasians, African American, and Hispanic cohorts, the average number of HLA class II molecular mismatches that were changed by imputation was greater than HLA class I loci mismatches; for Asian Americans, the average number of HLA class I and HLA class II imputation-induced changes were similar.

**TABLE 1.** Molecular mismatch analysis by single HLA loci using high-resolution and imputed HLA genotyping in racially concordant surrogate cohorts

HLA loci	No. of molecular mismatches		Pairwise difference between genotyping methods	No. of molecular mismatches		Pairwise difference between genotyping methods
	High-resolution genotyping	Imputed genotyping		High-resolution genotyping	Imputed genotyping	
	Mean ± SD	Mean ± SD	Mean ± SD	Mean ± SD	Mean ± SD	Mean ± SD
	African American			Asian American		
*A*	10.2 ± 5.3	9.9 ± 5.4	0.84 ± 1.00	9.4 ± 6.9	9.7 ± 6.8	0.61 ± 1.03
*B*	7.4 ± 4.0	7.7 ± 4.1	0.25 ± 0.84	7.7 ± 4.4	7.6 ± 4.4	0.15 ± 0.43
*C*	4.7 ± 3.5	4.8 ± 3.5	0.08 ± 0.33	7.1 ± 4.8	6.5 ± 4.7	0.59 ± 1.74
*DRB1*	10.6 ± 6.3	10.2 ± 6.2	1.05 ± 1.68	9.4 ± 5.3	9.5 ± 5.3	0.74 ± 0.87
*DRB345*	8.2 ± 6.0	7.2 ± 5.9	2.23 ± 3.18	9.9 ± 8.2	9.6 ± 8.5	1.32 ± 1.71
*DQB1*	12.8 ± 8.2	12.3 ± 8.0	0.67 ± 0.86	13.1 ± 7.8	12.8 ± 7.8	0.77 ± 1.23
*DQA1*	3.2 ± 2.7			4.3 ± 3.0		
*DPB1*	5.8 ± 3.3			5.4 ± 3.7		
*DPA1*	3.2 ± 3.0			0.9 ± 2.0		
	**Caucasian**			**Hispanic**		
*A*	10.0 ± 7.0	10.0 ± 7.0	0.00 ± 0.00	10.0 ± 4.6	9.7 ± 4.5	0.29 ± 0.64
*B*	7.1 ± 4.2	7.1 ± 4.2	0.00 ± 0.00	7.7 ± 4.0	7.8 ± 4.0	0.20 ± 0.51
*C*	4.0 ± 3.3	4.2 ± 3.3	0.15 ± 0.78	6.7 ± 4.0	6.7 ± 4.0	0.02 ± 0.14
*DRB1*	9.1 ± 6.3	9.2 ± 6.3	0.33 ± 0.70	9.2 ± 6.4	9.1 ± 6.7	1.42 ± 2.06
*DRB345*	7.8 ± 8.3	7.8 ± 8.5	0.46 ± 0.73	7.3 ± 7.7	7.0 ± 7.8	0.67 ± 1.06
*DQB1*	14.2 ± 9.9	14.1 ± 9.8	0.13 ± 0.44	11.9 ± 9.0	11.7 ± 9.0	0.28 ± 0.80
*DQA1*	3.9 ± 3.4			3.1 ± 2.4		
*DPB1*	5.2 ± 3.8			4.7 ± 4.1		
*DPA1*	1.0 ± 2.0			1.7 ± 2.4		

AVG and SD of the number of molecular mismatches using high-resolution and imputed genotyping for each racial cohort.

AVG, average.

### Total HLA Class II Epitope Mismatch Burden Is not Significantly Impacted by Imputation

We next evaluated the impact of imputation on molecular mismatch analysis for grouped HLA class I and class II loci. Imputation altered the sum of HLA class I molecular mismatches by a small number (range, 0.15–1.20), although it did reach statistical significance in the paired analysis of Caucasian and Hispanic cohorts (Table 2). Overall, the number of class II molecular mismatches obtained by each method was highly correlated (*R*^2^ = 0.91–0.95 for Asian Americans, Caucasians, and Hispanics) and was lowest for African Americans (*R*^2^ = 0.75; **Figure S1B, SDC,** http://links.lww.com/TXD/A666). The sum of HLA class II loci molecular mismatch has been shown to correlate with dnDSA formation and graft rejection in multiple studies.^13,15,18^ Imputation changed the average number of HLA class II molecular mismatches (HLA-DRB1, -DRB345, and -DQB1) by 0.71–2.16 within cohorts (Table 2). This difference was statistically significant for all groups except Caucasians (Table 2). Thus, in these racially concordant cohorts, the impact of imputation is small in magnitude for both class I and class II molecular mismatch analysis.

**TABLE 2.** Molecular mismatch analysis by HLA class and class II loci using high-resolution and imputed HLA genotyping in racially concordant surrogate cohorts

Donor-recipient pair racially concordant		No. of molecular mismatches		Pairwise difference between genotyping methods	
		High-resolution genotyping	Imputed genotyping		
		Mean ± SD	Mean ± SD	Mean ± SD	*P*
African American	Class I	22.3 ± 8.5	22.3 ± 8.8	0.95 ± 1.21	8.54E-01
	Class II	31.2 ± 13.9	29.3 ± 13.0	3.08 ± 3.90	8.32E-09
	Total	50.2 ± 13.4	51.6 ± 13.7	4.99 ± 4.87	4.65E-03
Asian	Class I	24.2 ± 11.0	23.9 ± 10.9	1.20 ± 2.11	8.48E-02
	Class II	32.4 ± 16.4	31.9 ± 16.5	1.95 ± 2.23	1.61E-02
	Total	54.3 ± 20.2	55.7 ± 20.1	4.17 ± 2.86	1.03E-03
Caucasian	Class I	21.1 ± 9.1	21.3 ± 9.1	0.15 ± 0.8	9.15E-03
	Class II	30.3 ± 19.4	30.4 ± 19.5	0.71 ± 1.0	3.89E-01
	Total	46.6 ± 19.4	48.4 ± 20.5	1.02 ± 1.50	3.45E-06
Hispanic	Class I	24.4 ± 7.4	24.1 ± 7.3	0.35 ± 0.69	3.76E-05
	Class II	28.3 ± 17.0	27.8 ± 17.5	2.16 ± 2.49	3.48E-02
	Total	52.3 ± 16.9	51.9 ± 19.2	2.87 ± 2.76	1.66E-01

AVG and SD of the number of molecular mismatches using high-resolution and imputed genotyping for each racial cohort.

AVG, average.

### Molecular Mismatch Risk Categorization Is Minimally Impacted by Imputation for Racially Concordant Donor-Recipient Pairs

**FIGURE 2.**
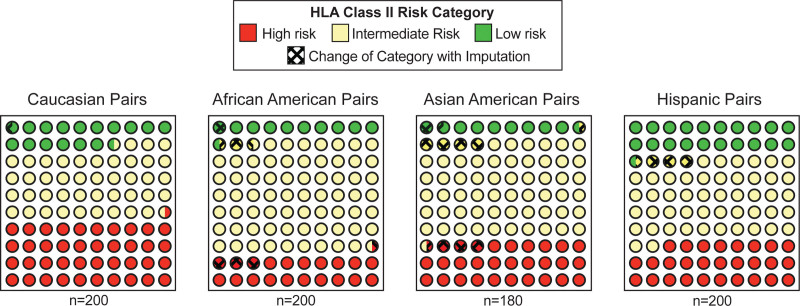
Impact of imputation on HLA class II risk category for racially concordant donor-recipient pairs. Part-of-whole analysis is depicted in which each dot represents 1% of pairs (n = 180–200 pairs/cohort). Solid colors depict risk category and cross-hatch indicates pairs for which imputation altered the risk category.

Recent studies have used molecular mismatch analysis to categorize donor-recipient pairs into high-, intermediate-, and low-risk groups.^15,20^ A central question regarding the clinical utility of imputation is whether the inaccuracy in molecular mismatch by imputation results in changes in risk categorization for donor-recipient pairs.^22,26-28^ We first evaluated the accuracy of imputation to categorize the molecular mismatch risk of each pair. We tabulated the number of times that the imputed molecular mismatch analysis changed the risk categorization of a donor-recipient pair (eg, from high to low risk or vice versa). For intermediate-risk pairs, we considered changes into either the high-risk or low-risk category. Overall, imputation preserved the high-resolution molecular mismatch risk category of 90.6%–99.5% of racially concordant pairs (Figure 2 and Table 3). As imputation had the smallest impact on risk category changes of the Caucasian cohort, we calculated the odds ratio (OR) of imputation to change the risk category for the other racial groups relative to Caucasians. African Americans and Asian American pairs, but not Hispanic pairs, had an increased OR for a risk category change relative to Caucasians (*P* = 0.018 African Americans, *P* = 0.003 Asian Americans). Overall, imputation preserved the correct molecular mismatch risk categorization for racially concordant pairs in nearly all cases. However, there was an increased risk of incorrect classification for certain racial groups relative to Caucasians.

**TABLE 3.** Impact of imputation on racially concordant surrogate donor-recipient pairs

Racially concordant donor-recipient pair		Molecular mismatch risk group			% Pairs changed	Odds ratio	95% CI	*P*
		High	Intermediate	Low				
Caucasian	No. of pairs high-resolution typing	81	86	33				
	No. of pairs changed with imputation	0	0	1	0.5%	NA	NA	NA
African American	No. of pairs high-resolution typing	41	138	21				
	No. of pairs changed with imputation	7	4	2	6.5%	13.8	1.8-106.6	0.012
Hispanic	No. of pairs high-resolution typing	56	103	41				
	No. of pairs changed with imputation	0	7	0	3.5%	7.2	0.9-59.1	0.065
Asian American	No. of pairs high-resolution typing	53	110	17				
	No. of pairs changed with imputation	6	8	3	9.4%	20.8	2.7-157.4	0.003

Number of pairs in each HLA class II molecular mismatch risk group are shown for each racially concordant cohort. Odds ratio calculated vs Caucasian cohort.

CI, confidence interval.

### Molecular Mismatch Risk Categorization Is Minimally Impacted by Imputation for Racially Discordant Donor-Recipient Pairs

**FIGURE 3.**
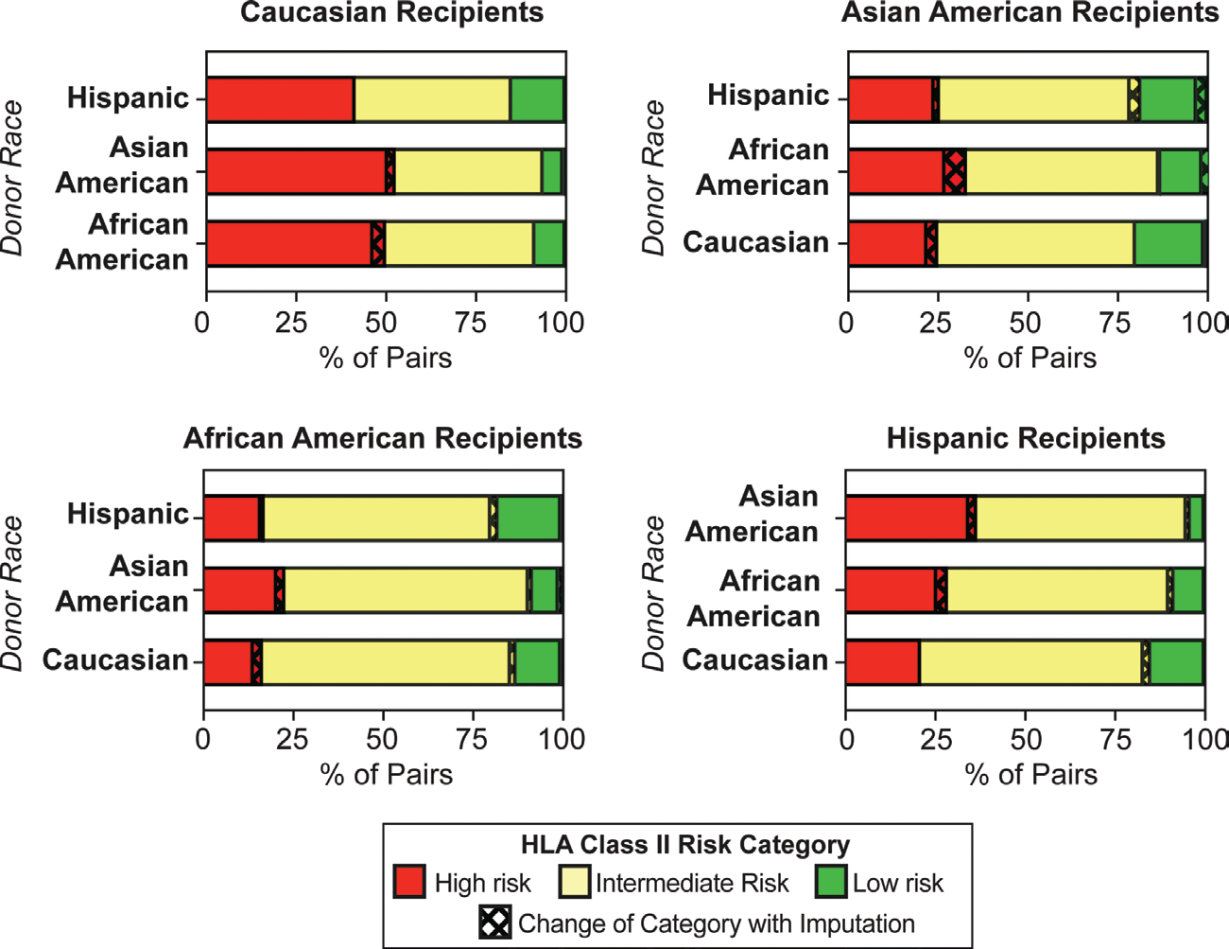
Impact of imputation on HLA class II risk category for racially discordant donor-recipient pairs. Part-of-whole analysis is depicted for each bar (n = 180–200 pairs/cohort). Solid colors depict the frequency of pairs in each risk category and cross-hatch indicates pairs for which imputation altered the risk category.

We next evaluated whether the changes induced by imputation impacted the molecular mismatch risk category for racially discordant pairs. We performed molecular mismatch analysis on the high-resolution or imputed HLA genotyping of racially discordant pairs using each combination of donor-recipient race (Table 4 and Figure 3). There was overall a strong correlation between the number of molecular mismatches obtained via high-resolution genotyping and imputation (class I *R*^2^ = 0.84–0.99, class II *R*^2^ = 0.82–96; **Figure S1, SDC,** http://links.lww.com/TXD/A666). We found that overall, the molecular mismatch risk categorization did not change for 91.5%–100% of racially discordant pairs. Among recipient groups, imputation had a slightly greater impact on Asian Americans as the risk categorization changed, with 7.5% for Hispanic donors and 8.5% for African American donors, respectively. The other recipient groups appeared to have similar impact imputation on molecular mismatch risk, regardless of the donor race, with a range of 2.0%–4.5%. One exception is Caucasian recipients with Hispanic donors, for which imputation had no impact on molecular mismatch risk categorization. To assess the impact of donor race on the effect of imputation, we calculated the OR of racially discordant donors relative to the racial concordant pairs. We found that donor race did not significantly impact the changes in the molecular mismatch risk categorization with imputation (Table 4). Among all combinations, only Asian American-Caucasian recipient-donor pairs were statistically significant (*P* = 0.038). Overall, the results demonstrate that imputation does not generate large changes to the molecular mismatch risk categorization of racially discordant donor-recipient pairs.

**TABLE 4.** Impact of imputation on racially discordant surrogate donor-recipient pairs

Racially discordant donor-recipient pairs			Molecular mismatch risk group			% Pairs changed	Odds ratio	95% CI	*P*
Recipient race	Donor race								
			High	Intermediate	Low				
African American	Caucasian	No. of pairs high-resolution typing	33	141	26	4.5%	0.7	0.3-1.6	0.38
		No. of pairs changed with imputation	5	3	1				
	Asian	No. of pairs high-resolution typing	41	124	15	4.4%	0.7	0.3-1.7	0.38
		No. of pairs changed with imputation	4	2	2				
	Hispanic	No. of pairs high-resolution typing	34	130	36	3.5%	0.5	0.2-1.3	0.18
		No. of pairs changed with imputation	2	4	1				
Asian American	Caucasian	No. of pairs high-resolution typing	50	110	40	4.0%	0.4	0.2-0.9	0.038
		No. of pairs changed with imputation	6	0	2				
	African American	No. of pairs high-resolution typing	66	107	27	8.5%	0.9	0.4-1.8	0.75
		No. of pairs changed with imputation	12	1	4				
	Hispanic	No. of pairs high-resolution typing	51	112	37	7.5%	0.8	0.4-1.6	0.50
		No. of pairs changed with imputation	3	6	6				
Caucasian	African American	No. of pairs high-resolution typing	100	83	17	3.5%	7.2	.9-59.2	0.07
		No. of pairs changed with imputation	7	0	0				
	Asian	No. of pairs high-resolution typing	95	74	11	2.8%	5.7	0.7-49.1	0.11
		No. of pairs changed with imputation	4	0	1				
	Hispanic	No. of pairs high-resolution typing	83	87	30	0.0%	0	0-Inf.	0.99
		No. of pairs changed with imputation	0	0	0				
Hispanic	Caucasian	No. of pairs high-resolution typing	42	128	30	2.0%	0.6	0.2-2.0	0.37
		No. of pairs changed with imputation	0	4	0				
	African American	No. of pairs high-resolution typing	57	126	17	4.5%	1.3	0.5-3.6	0.61
		No. of pairs changed with imputation	6	3	0				
	Asian	No. of pairs high-resolution typing	66	107	7	3.3%	1	0.3-2.9	0.93
		No. of pairs changed with imputation	4	2	0				

CI, confidence interval.

### Molecular Mismatch Risk Categorization Is Minimally Impacted by Imputation in a Cohort of Deceased Donor Kidney Transplant Patients

To evaluate the impact of the imputation of molecular mismatch risk categorization in transplant patients, we next evaluated consecutive kidney transplants at Johns Hopkins Hospital in 2022. To maximize the HLA disparity between the donors and recipients, we selected 35 patients who received deceased donor allografts and had a cPRA of ≤80. This cohort was racially diverse, as it comprised Caucasian, African American, Asian American, and Hispanic patients and donors (Table 5; **Table S1, SDC,** http://links.lww.com/TXD/A666). Of 35 cases, in 25 (71%), the donor-recipient racial group was discordant (Table 5; **Table S1, SDC,** http://links.lww.com/TXD/A666). In the same manner as the surrogate pairs described previously, we performed molecular mismatch analysis on the high-resolution genotyping and low-resolution genotyping imputed with HaploStats. The number of molecular mismatches obtained by each genotyping method was highly correlated (class I *R*^2^ = 1.00, class II *R*^2^ = 0.97; **Figure S2, SDC,** http://links.lww.com/TXD/A666). We found that imputation for the kidney transplant cohort changed the molecular mismatch risk categorization of only 1 pair (2.9% of the total) from low to intermediate risk (Table 6). Thus, imputation did not significantly affect the clinical risk stratification based on molecular mismatch analysis in this deceased donor kidney transplant cohort.

**TABLE 5.** Racial group identification of kidney transplant cohort

Recipient racial group	No. of recipients	Racially concordant donors	Racial discordant donors
African American	16	2	14
Asian	4	0	4
Caucasian	10	8	2
Hispanic	5	0	5
Total	35	10	25

Number of kidney transplant patients from each racial group for whom molecular mismatch analysis was performed.

**TABLE 6.** Molecular mismatch risk categorization resulting from high-resolution or imputed genotyping of renal transplant cohort

Donor-recipient pair racially concordant	Molecular mismatch risk group			% Pairs changed
	High	Intermediate	Low	
No. of pairs high-resolution typing	3	21	11	
No. of pairs changed with imputation	0	0	1	2.9%

## DISCUSSION

In the current era of solid organ transplantation, molecular genotyping technology and highly sensitive HLA antibody analysis allow for DSA-free donors to be selected.^30-32^ Whereas physical crossmatching identified moderate-strength antibodies, low-level DSAs can now be actively avoided in most cases at the time of transplant. However, in the decade after the transplant, approximately 20% of renal transplant patients develop antidonor HLA antibodies and are at an increased risk of graft failure.^6,20,33^ Although HLA antigen mismatch correlates with outcomes in large cohorts, antigen-level mismatch does not provide strong prognostic information at the time of transplant.^34,35^ This has focused efforts on identifying the minority of patients who are at increased risk of posttransplant dnDSA formation to prolong graft survival.

The evaluation of HLA compatibility based on antigen-level (whole protein) differences has been the backbone of transplantation for >50 y. However, the biochemical sites on which antibody recognition HLA proteins, called epitopes or eplets, have also been long recognized. For example, antibodies specific for epitopes that are common across multiple HLA allele groups are routinely considered in clinical antibody assessment. Duquesnoy^36^ took this concept further by beginning a catalog of predicted and known HLA epitopes. Seminal work by Wiebe and Nickerson^19^ first evaluated whether epitope-level donor-recipient mismatching would provide a better immunologic risk assessment than whole HLA protein assessment. The quantification of HLA epitope mismatches, now termed molecular mismatch analysis, was found to correlate with posttransplant outcomes in a cohort of renal transplant recipients. This work has evolved to show that the number of HLA class II molecular mismatches could be used to retrospectively stratify patients into high-, intermediate-, and low-risk categories for dnDSA formation after transplant.

The ultimate clinical utility of molecular mismatch analysis in a prospective real-world clinical setting remains undetermined. Molecular mismatch analysis is undergoing evaluation as a biomarker by the Food and Drug Administration and there is widespread consensus that multicenter and prospective clinical trials are needed.^13^ From an informatics perspective, molecular mismatch analysis is logistically feasible within the context of clinical evaluation of deceased donors. In addition, there is growing interest in performing retrospective outcomes analysis of transplant populations (including other solid organ transplant populations) using molecular mismatch analysis.

A major question for these applications is how imputation impacts the results of molecular mismatch analysis. The minimum requirement for HLA genotyping for solid organ transplantation is at the HLA antigen level, and the pace of deceased donor HLA genotyping currently requires low-resolution genotyping methods. Although sequencing methods are rapidly evolving, the vast majority of routine donor and recipient HLA genotyping performed for solid organ transplantation is intermediate or low resolution. Thus, for individual institutions to study the impact of molecular mismatch analysis on patient populations or for the study of multicenter retrospective analyses, imputation to high-resolution genotyping is required.

Two recent studies evaluated the impact of imputation in different cohorts. Engen et al^23^ evaluated the impact of HaploStats imputation on the assignment of whole HLA haplotypes and found that imputation introduced inaccuracies in approximately 36.5%–55% of cases (depending on race). This study used a racially diverse cohort but focused on the analytic accuracy of imputation on full HLA haplotype assignment and did not systematically compare the impact of imputation on molecular mismatch analysis between donor-recipient pairs. Senev et al^37^ analyzed a cohort of 1000 kidney transplant recipients with low-resolution and high-resolution genotyping. They found that imputation changed the HLA class II molecular mismatch analysis in 45.1% of cases but did not evaluate the impact of molecular mismatch risk categorization in their cohort.

We wanted to extend these conclusions about the inaccuracies that imputation introduces into HLA genotyping to assess its impact on clinical utility, specifically the molecular mismatch risk assessment. In racially concordant and discordant surrogate pairs, we found that imputation introduced changes to the molecular mismatch analysis in both racially concordant and discordant cohorts. This result is consistent with the analysis of Engen et al who showed that imputation introduced errors in more than half of their cohort. However, when we analyzed the number of pairs that had the risk categorization altered by imputation, we found that 90.6%–99.5% of racially concordant pairs and 91.5%–100% of racially discordant pairs were correctly classified. Importantly, no major effect was observed by donor or recipient race.

Finally, to validate these findings using surrogate pairs, we conducted similar analyses in a cohort of patients who received a renal transplant at our center. We selected consecutive transplants from deceased donors and filtered outpatients with very high cPRA values (>80%) to avoid high levels of HLA matching that would theoretically minimize the molecular mismatch between donors and recipients. This cohort was racially diverse and comprised of 72% racially discordant pairs. When we performed molecular mismatch analysis on high-resolution and imputed genotyping, we found that imputation only altered the molecular mismatch risk categorization of 1 pair out of 35 (2.9%).

Together, these results provide evidence that molecular mismatch risk categorization using imputed low-resolution genotyping is highly accurate. For deceased donor or living donor renal transplantation in which low-/intermediate-resolution HLA genotyping is available for donor and/or recipient, these results show that an accurate molecular mismatch risk categorization can be obtained. We found that imputation does introduce inaccuracies at the individual HLA loci, which is in line with other recent reports considering the effect of imputation on haplotype prediction.^23,37^ Although this limits its use for specific clinical purposes, such as donor-specific antibody identification or organ allocation, each downstream use of molecular mismatch analysis has different goals and should not be conflated. Further investigation is certainly needed to fully incorporate molecular mismatch analysis into clinical practice or organ allocation algorithms. In particular, the definitions of high-, intermediate-, and low-risk molecular mismatch categories have been generated from only a few transplant patient cohorts and should be correlated with clinical outcome (graft rejection and development of dnDSAs) in additional studies.

There are several limitations to this study. We used broad racial groups that relied on self-identification and could not use more detailed ethnic groups within these broader categories.^25^ Racial and ethnic identification is complex, and there are many social and cultural factors that can impact self-identified race. As our study is US-based, not all racial identities are applicable in other regions. When performing imputation, we used the most likely high-resolution genotype result, and we did not account for the imputation prediction likelihoods or the remaining imputation prediction distribution for the donor-recipient pairs. These considerations were also not accounted for in the prior studies evaluating imputation work by Engen et al^23^ or Senev et al.^37^ As low-resolution HLA input for imputation usually results in a large prediction distribution,^22,24,25^ a multiple imputation approach based on random sampling from the entire prediction distribution may further improve the accuracy of imputation to predict HLA genotyping from low-resolution input.^38^ Future studies are needed to explore the impact of the most likely versus multiple imputations on molecular mismatch analysis results. In addition, although our surrogate pair analysis included systematic evaluation of racially concordant and discordant pairs, we relied on a modest number of genotypes to produce cohorts of 180–200 pairs. Additional validation studies are needed to corroborate these findings in larger cohorts of racially diverse donors and patients.

Overall, the results of this study provide important evidence that high-resolution genotyping is not necessary to accurately perform molecular mismatch analysis to categorize patients into immunological risk groups. Although advances in next-generation sequencing platforms will enable high-resolution HLA genotyping for all transplantation in the future, the use of low- and intermediate-resolution genotyping remains a reality for the field for multiple years to come. HLA antigen matching and molecular mismatch analysis are only part of a multifactorial process that is used to select organs for each transplant candidate. This study supports the use of imputation in the performance of clinical research studies to evaluate molecular mismatch analysis.

## ACKNOWLEDGMENTS

The authors thank members of the Johns Hopkins Immunogenetics Laboratory for performing clinical testing and Dr Dan Brennan for critical reading of the article.

## Supplementary Material


