# Supplementary material for: HLA Genotype Imputation Results in Largely Accurate Epitope Mismatch Risk Categorization Across Racial Groups
Source: Transplant Direct. 2024 Jun 20;10(7):e1639. doi: 10.1097/TXD.0000000000001639 (PMC11191912; doi:10.1097/TXD.0000000000001639)
Supplement: Supplementary file 1 [file txd-10-e1639-s001.pdf]

Supplemental Information

Table of Contents

1. Table S1
2. Figure S1
3. Figure S2

Pair No.	cPRA	Pt Race	Donor Race
1	38%	African American	Hispanic
2	0%	Caucasian	Caucasian
3	26%	Hispanic	Caucasian
4	31%	Hispanic	Caucasian
5	0%	Caucasian	African American
6	0%	African American	Hispanic
7	7%	African American	Caucasian
8	0%	African American	African American
9	80%	African American	Hispanic
10	75%	African American	African American
11	54%	African american	Caucasian
12	0%	Caucasian	Caucasian
13	35%	Caucasian	Caucasian
14	37%	Hispanic	African American
15	80%	Asian	Caucasian
16	0%	Caucasian	Caucasian
17	67%	African American	Hispanic
18	0%	African American	Hispanic
19	65%	African American	Hispanic
20	74%	Caucasian	Hispanic
21	63%	African American	Caucasian
22	0%	Caucasian	Caucasian
23	0%	African American	Caucasian
24	0%	Asian	Caucasian
25	0%	African American	Hispanic
26	50%	Caucasian	Caucasian
27	0%	Asian	Hispanic
28	27%	African American	Caucasian
29	37%	Caucasian	Caucasian
30	50%	Hispanic	Caucasian
31	68%	African American	Caucasian
32	26%	African American	Caucasian
33	53%	Caucasian	Caucasian
34	0%	Asian	Caucasian
35	0%	Hispanic	African American

Table S1. Racial group and cPRA of patients and racial group of donors included in deceased donor renal transplant cohort.

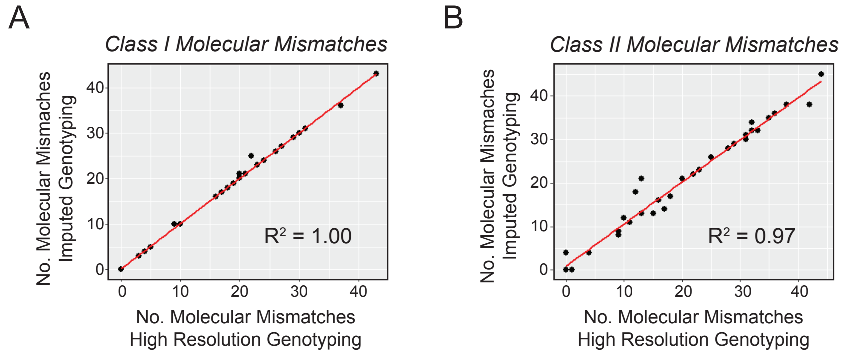

Figure S1. The number of molecular mismatches calculated by imputation and high-resolution genotyping for surrogate pairs. (A) HLA Class I or (B) HLA Class II linear regression analysis of the number of molecular mismatches calculated using imputed or high-resolution genotyping for surrogate pairs that are racially concordant or discordant.

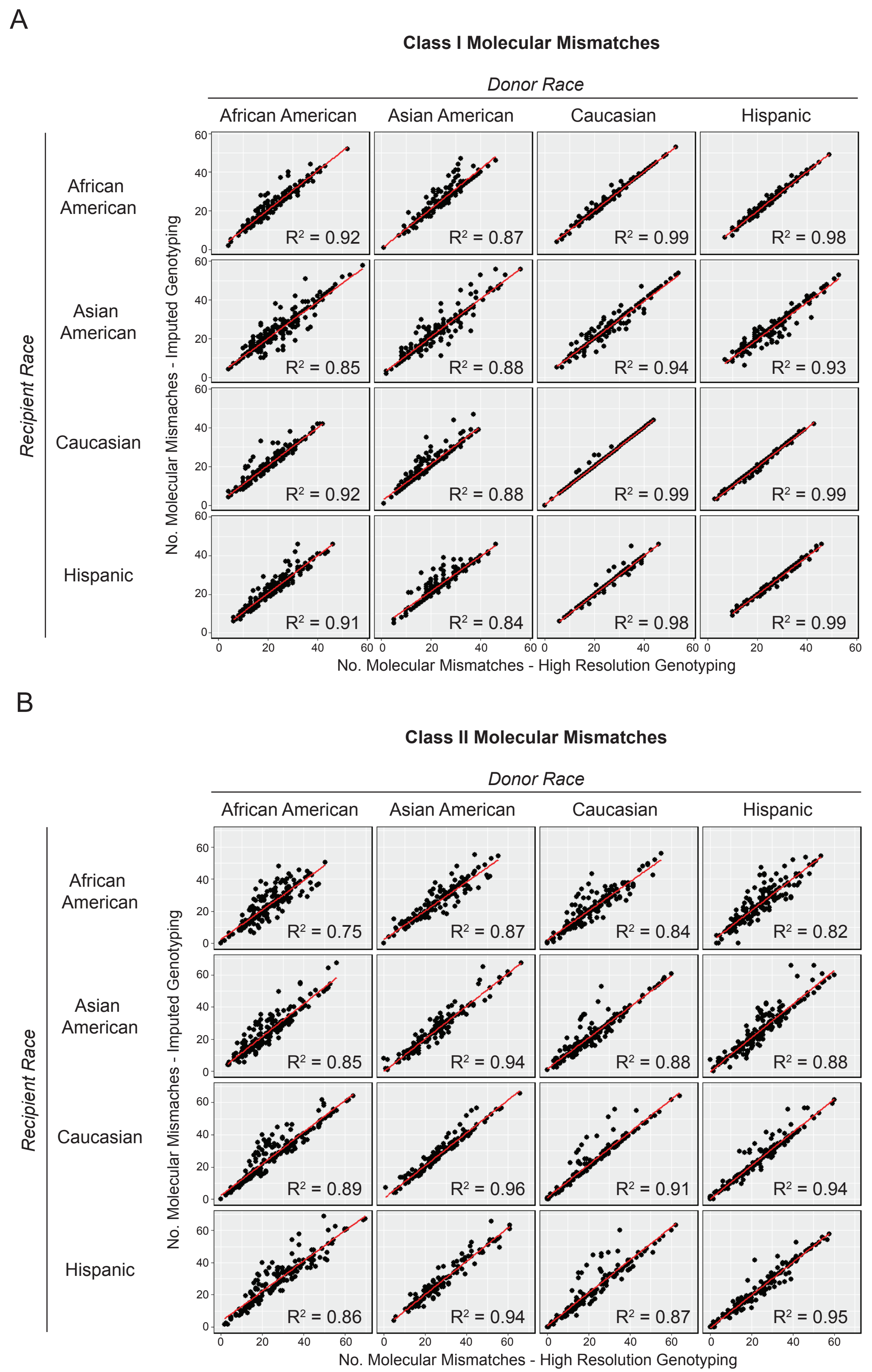

Figure S2. The number of molecular mismatches calculated by imputation and high-resolution genotyping for renal transplant pairs. Linear regression analysis of the number of molecular mismatches calculated using imputed or high-resolution genotyping for surrogate pairs that are racially concordant or discordant.
